# Comparison of a Bioresorbable, Magnesium-Based Sirolimus-Eluting Stent with a Permanent, Everolimus-Eluting Metallic Stent for Treating Patients with Acute Coronary Syndrome: the PRAGUE-22 Study

**DOI:** 10.1007/s10557-021-07258-z

**Published:** 2021-09-10

**Authors:** Petr Toušek, Tomáš Lazarák, Ivo Varvařovský, Markéta Nováčková, Marek Neuberg, Viktor Kočka

**Affiliations:** 1 Department of Cardiology, Third Medical Faculty Charles University and University Hospital Královské Vinohrady, Prague, Czech Republic; 2 Department of Cardiology, AGEL, Pardubice, Czech Republic; 3 Medtronic Czechia, Partner of INTERCARDIS Project, Prague, Czech Republic

**Keywords:** Acute coronary syndrome, Percutaneous coronary intervention, Magnesium-based bioresorbable stents, Late lumen loss, Outcome

## Abstract

**Background:**

Magnesium-based bioresorbable Magmaris stents are rapidly resorbed. Few randomized studies have evaluated the efficacy of such stents in patients with acute coronary syndrome.

**Aim:**

To investigate late lumen loss as assessed via quantitative coronary angiography (QCA) and optical coherence tomography (OCT) in patients with acute coronary syndrome treated with Magmaris stents or permanent, everolimus-eluting metallic Xience stents.

**Methods and Results:**

This PRAGUE-22 study was a two-centre, investigator-initiated, randomized study. Fifty patients were randomized based on the inclusion criteria for acute coronary syndrome and the anatomical suitability to receive Magmaris or Xience stents. The patient characteristics did not differ between the Magmaris group (*n* = 25) and Xience group (*n* = 25). The mean ages were 57.0 ± 10.5 vs. 55.5 ± 9.2 years (*p* = 0.541) and the total implanted stent length was 24.6 ± 10.7 mm vs. 27.6 ± 11.1 mm (*p* = 0.368), respectively. Four clinical events occurred in the Magmaris group and one in the Xience group during 12 months of follow-up. The extent of late lumen loss (assessed via QCA) at 12 months was greater in the Magmaris group than in the Xience group (0.54 ± 0.70 vs. 0.11 ± 0.37 mm; *p* = 0.029). The late lumen loss diameter (measured via OCT) in the Magmaris group was also significantly larger than that in the Xience group (0.59 ± 0.37 vs. 0.22 ± 0.20 mm; *p* = 0.01).

**Conclusion:**

Implantation of a magnesium-based bioresorbable stent in patients with acute coronary syndrome is associated with a greater extent of late lumen loss at the 12-month follow-up compared with implantation of a permanent, everolimus-eluting metallic stent.

**Trial Registration:** ISRCTN89434356

## Introduction

In 2011, interventional cardiologists enthusiastically welcomed the first generation of polymeric bioresorbable stents. However, the Absorb stent (Abbott Vascular, Santa Clara, CA, USA) was associated with a higher risk of target lesion revascularization and stent thrombosis compared with drug-eluting metallic stents in randomized trials [1, 2] and in an acute myocardial infarction setting [3]. Furthermore, resorption only took place more than 3 years after implantation, being complete at 5 years [4, 5]. Magnesium-based bioresorbable stents represent different technology with quicker resorption process and are sometimes viewed as second-generation bioresorbable stents. Early studies reported good clinical results for up to 3 years after implantation in patients with stable coronary artery disease (simple lesions) [6, 7]. However, the first randomized study in patients with ST-elevation acute myocardial infarctions reported a lower angiographic efficacy and a higher rate of target lesion failure (TLF) after the implantation of magnesium-based bioresorbable stents compared to the implantation of sirolimus-eluting metallic stents at the 12-month follow-up [8]. We investigated the 12-month efficacy of bioresorbable, magnesium-based sirolimus-eluting stents compared to everolimus-eluting metallic stents in patients with acute coronary syndrome.

## Methods

This was a two-centre, investigator-initiated academic randomized study. Patients were randomized using the envelope method between May 2017 and December 2019 into Magmaris stent- (Biotronic AG, Bulach, Switzerland) and Xience stent-treated (Abbott, Santa Clara, CA, USA) groups. The inclusion criteria were ST-elevation myocardial infarction (STEMI) within 24 h of symptom onset, non-ST elevation myocardial infarction (non-STEMI), or unstable angina caused by thrombotic acute coronary stenosis, and a coronary artery diameter appropriate for implantation of either stent (vessel diameter 2.7 to 3.7 mm). The exclusion criteria were cardiogenic shock, pulmonary oedema, expected survival < 3 years because of severe comorbidities, any contraindication for 12 months of dual antiplatelet treatment (including peroral anticoagulants), any diffuse calcification or extreme tortuosity of the target vessel, in-stent restenosis or stent thrombosis as the culprit lesion, and left main vessel stenosis. The study was approved by the ethics committee of each centre and by our national multicentric ethics committee, and written informed consent was obtained from all patients. The study adhered to the tenets of the Declaration of Helsinki.

### Procedural Technique

The treating physicians had considerable experience with bioresorbable stents and adhered to a standardized technique that accounted for stenosis predilation using a balloon of diameter 0.5 mm or less than that of the vessel and confirmed the correct sizing and postdilation of the implanted stent using a noncompliant balloon inflated to > 16 atmospheres of the same nominal size as the scaffold implantation balloon or up to 0.5 mm larger. The implantation of metallic Xience stents was left to the discretion of the physicians, with postdilation strongly recommended. Optical coherence tomography (OCT) of acute-phase patients was recommended but not mandatory (the most common reasons for omission were haemodynamic instability, clinically significant arrhythmia, and lack of an available OCT catheter). The use of the same type of stent was recommended if a second (nontarget) lesion were to be treated.

### Follow-up and Study Endpoints

**Fig. 1:**
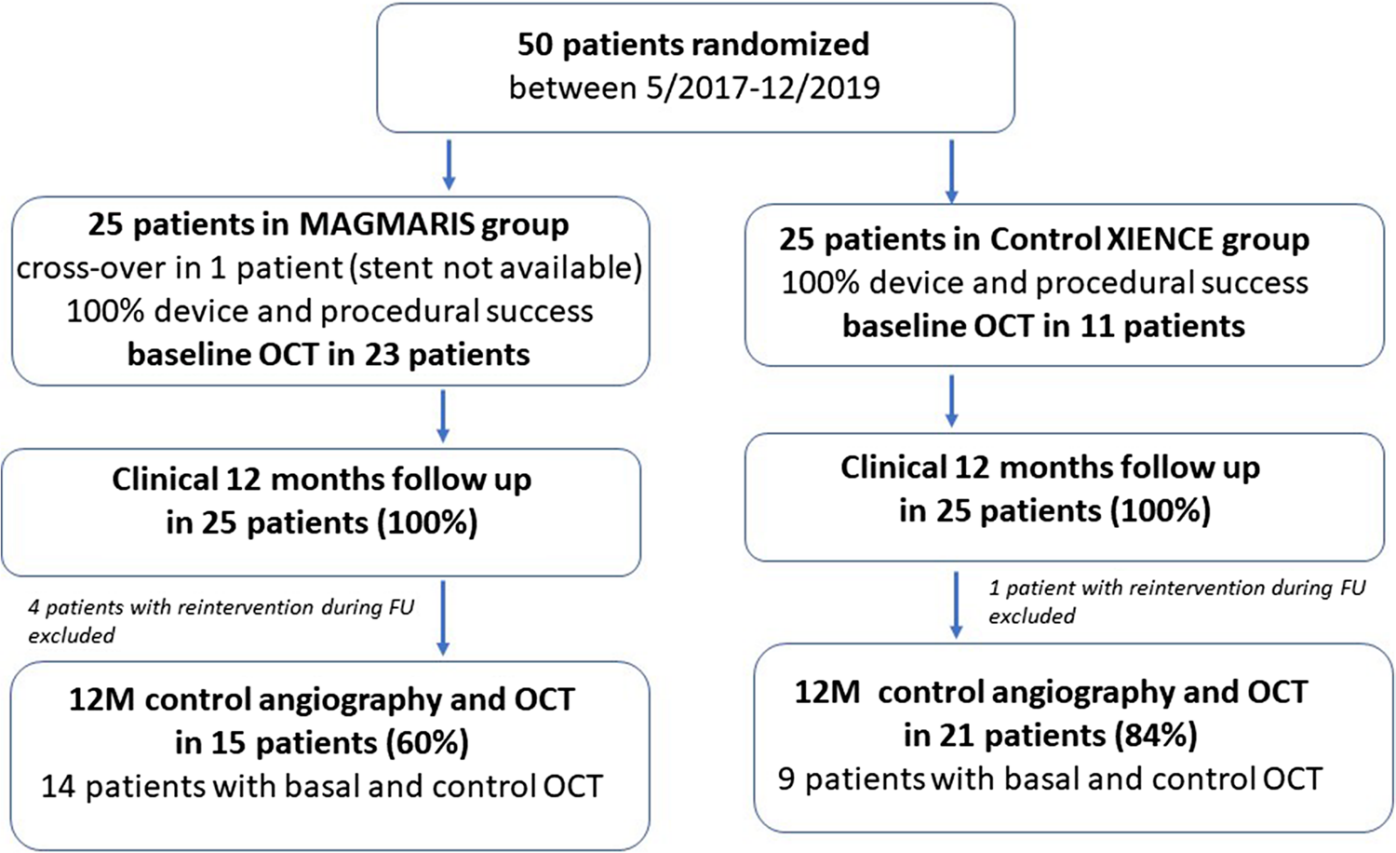
Patient enrolment and follow-up data showing the numbers of patients who underwent control angiography and optical coherence tomography (OCT)

The protocol mandated dual antiplatelet therapy for 12 months after the implantation of either stent. All patients were examined at 1 and 6 months by blinded cardiologists. Repeat quantitative coronary angiography (QCA) and OCT were performed at 12 months. Figure 1 shows the numbers of patients who underwent clinical follow-up and coronary artery imaging at 12 months. The primary endpoints of our study were late lumen loss evident at the 12-month follow-up as assessed by QCA and OCT. The secondary endpoints were the device and procedural success rates, combined clinical endpoints (death, stent thrombosis, target vessel myocardial infarction), clinical TLF rate, Magmaris stent resorption status, and qualitative assessment of healing in both groups.

### QCA

The treated segments (5 mm proximal and distal to the scaffold edge) were analyzed using Philips IntelliSpace cardiovascular software (Philips Medical Systems, Eindhoven, the Netherlands). Image calibration was performed using a contrast-filled guiding catheter. The following QCA parameters were measured after stent implantation in both groups: proximal and distal reference vessel diameters (RVDs)—defined as those of the largest lumina within 5 mm of the proximal and distal edges, the minimal lumen diameters (MLDs) within the scaffolds, and the mean scaffold diameters. The stenosis diameter was calculated from the MLD, and the average of the proximal and distal RVDs and was expressed as a percentage. The extent of late lumen loss was represented as the MLD at baseline minus the MLD at follow-up. Binary restenosis was defined as a stenosis diameter > 50% on follow-up QCA.

### OCT

OCT of infarct-related vessels with stents was performed using the frequency-domain C7 system and a Dragonfly or Dragonfly Duo catheter (St. Jude Medical, St. Paul, MN, USA). The pullback speed was 20 mm/s, and the image acquisition rate was 100 or 200 frames/s. OCT measurements were performed using commercial software for offline analysis (Ilumien Optis system; LightLab Imaging, Westford, MA, USA). The OCT results were analyzed on a per-patient and per-frame basis. The reference vessel area and diameter were measured at the sites with the largest lumina within 5 mm of the proximal and distal scaffold edges. Stent area/diameter and lumen area/diameter at follow-up were measured using vessel cross-sections at 1-mm intervals. The mean stent/lumen area and diameter were defined as the means of those of all cross-sections within the device. The minimal luminal area was the smallest area within the device. QCA and OCT were performed by a single experienced unblinded operator (PT); qualitative OCT assessment of healing and neoatherosclerosis was conducted by two operators working in consensus. For qualitative analysis, we used the recent methodology of Gomez-Lara [9]. Depending on the highest numbers of cross-sections exhibiting one of four possible bioresorption/healing profiles, the outcomes of using both devices were classified into the following groups: struts indiscernible, visible struts completely integrated into the vessel wall, visible struts protruding into the lumen (causing characteristic bumps), and visibly protruding struts malapposed to the vessel wall. Neoatherosclerosis apparent on follow-up OCT was defined as the presence of at least one of the following findings between the stent and lumen or < 200 μm from the end-luminal border when struts were indiscernible: a fibrocalcific plaque (a signal-poor region with sharply delineated upper and lower borders), lipid-rich plaques (diffusely bordered, signal-poor regions), or signs of neovascularization [10].

### Statistical Analysis

Standard descriptive statistics were use; categorical variables are presented as absolute values and relative frequencies, whereas continuous variables are described as means with standard deviations. The significance of between-group differences was computed using Fisher’s exact test for two categorical variables, or the maximum likelihood *χ*^2^ test for variables with more than two categories. The significance of changes in continuous variables (QCA and OCT data) between baseline and follow-up was evaluated using the paired *t*-test. *P*-values < 0.05 were considered to indicate statistical significance. All analyses were performed with the aid of SPSS ver. 26.0.0.0 (IBM Corp., Armonk, NY, USA).

## Results

Fifty patients were enrolled; 25 patients were initially assigned to each group. There was one crossover from the Magmaris group, as a stent of the required length was not available and a Xience stent was thus placed. Table 1 summarizes the patient and procedural characteristics. There were no significant between-group differences with the exceptions of the lower lesion predilation and stent postdilation rates in the control group. Twelve-month clinical follow-up data were available for all 50 patients, and 15 patients in the Magmaris group and 21 in the control group agreed to undergo repeat QCA and OCT.

**Table 1:** Patient and procedural characteristics after randomization

		Magmaris group (*n* = 25)	Xience group (*n* = 25)	*P*-value
Male sex		16 (64%)	19 (76%)	0.27
Age (years ± SD)		57.0 ± 10.5	55.5 ± 9.2	0.54
BMI (kg/m^2^ ± SD)		28.5 ± 4.3	29.7 ± 4.6	0.66
EF (% ± SD)		55.8 ± 10.0	55.8 ± 8.4	0.90
Smokers		18 (72%)	14 (56%)	0.19
Diabetes		3 (12%)	8 (32%)	0.09
Diagnosis	STEMI	18 (72%)	14 (56%)	
	Non-STEMI	6 (24%)	7 (28%)	
	UAP	1 (4%)	4 (16%)	0.31
P2Y12	Tricagrelor	16 (64%)	21 (87.5%)	
	Prasugrel	4 (16%)	3 (12.5%)	
	Clopidogrel	5 (20%)	0 (0%)	0.055
Procedural characteristics				
Target vessel	LAD	11 (44%)	13 (52%)	
	LCx	5 (20%)	5 (20%)	
	RCA	9 (36%)	7 (28%)	0.66
Vessel disease	1	15 (60%)	14 (56%)	
	2	10 (40%)	9 (36%)	
	3	0 (0%)	2(8%)	0.35
Postdilation		23 (92%)	18 (72%)	*0.0069*
Predilation		25 (100%)	20 (80%)	*0.028*
No. of stents	1	21 (84%)	19 (76%)	
	2	3 (12%)	5 (20%)	
	3	1 (4%)	1 (4%)	0.74
Total stent length (mm)		24.6 ± 10.7	27.6 ± 11.1	0.37

*SD* standard deviation, *BMI* body mass index, *EF* ejection fraction, *STEMI* ST-elevation myocardial infarction, *UAP* unstable angina pectoris, *LAD* left anterior descending artery, *LCx* left circumflex artery, *RCA* right coronary artery

### QCA Results

The baseline and follow-up QCA characteristics of patients in both groups are summarized in Table 2. There were no differences in the baseline MLD and baseline mean in-stent lumen diameter between the groups. At 12 months, the late lumen loss was larger in the Magmaris group than that in the control group (0.54 ± 0.70 vs. 0.11 ± 0.37 mm; *p* = 0.029). The stented segment stenosis increased significantly in the Magmaris group during follow-up based on the baseline and 12-month QCA results (7.00 ± 8.41% vs. 27.05 ± 20.58%; *p* < 0.001) but not in the Xience group (10.16 ± 8.73% vs. 15.52 ± 11.62%; *p* = 0.141).

**Table 2:** QCA data

Magmaris group			
QCA analysis	Baseline QCA (*n* = 25)	Follow-up QCA (*n* = 15)	*P*-value
Minimal lumen diameter (mm ± SD)	2.47 ± 0.37	1.92 ± 0.57	*0.001*
Mean in-stent lumen diameter (mm ± SD)	2.64 ± 0.39	2.38 ± 0.48	0.077
Proximal RVD (mm ± SD)	2.81 ± 0.39	2.79 ± 0.46	0.85
Distal RVD (mm ± SD)	2.57 ± 0.45	2.57 ± 0.49	0.99
Mean RVD (mm ± SD)	2.65 ± 0.38	2.66 ± 0.41	0.91
Stenosis (% ± SD)	7.00 ± 8.41	27.05 ± 20.58	*0.001*
Xience group (control)			
QCA analysis	Baseline QCA (*n* = 25)	Follow-up QCA (*n* = 21)	*P*-value
Minimal lumen diameter (mm ± SD)	2.54 ± 0.39	2.43 ± 0.49	0.42
Mean in stent lumen diameter (mm ± SD)	2.84 ± 0.40	2.74 ± 0.45	0.43
Proximal RVD (mm ± SD)	2.94 ± 0.45	2.94 ± 0.49	0.99
Distal RVD (mm ± SD)	2.73 ± 0.39	2.73 ± 0.38	0.97
Mean RVD (mm ± SD)	2.84 ± 0.41	2.84 ± 0.41	0.95
Stenosis (% ± SD)	10.16 ± 8.73	15.52 ± 11.62	0.14

*QCA* quantitative coronary angiography, *RVD* reference vessel diameter, *SD* standard deviation

### OCT Results

One OCT dataset in the Magmaris group could not be subjected to final analysis because the image quality was poor. Also, four patients in the Magmaris group were excluded from the 12-month analysis because of TLF re-interventions featuring the implantation of permanent, drug-eluting metallic stents. Ultimately, 12-month follow-up OCT data on 14 patients in the Magmaris group and 21 patients in the Xience group were available. Of these, all 14 patients in the Magmaris group and 9 patients in the Xience group had undergone baseline OCT after stent implantation. Table 3 compares the OCT measurements between the groups at the 12-month follow-up, and the baseline and follow-up measurements of each group. The late lumen loss diameter was significantly larger in the Magmaris group compared to the Xience group (0.59 ± 0.37 vs. 0.22 ± 0.20 mm; *p* = 0.01). Qualitative assessment at the 12-month follow-up revealed that struts were indiscernible in 9 (60%) patients in the Magmaris group, visible struts were completely integrated into the vessel wall in 3 (20%) patients in the Magmaris group and 17 (81%) in the control group, and visible struts protruding into the lumen were apparent in 3 (20%) patients in the Magmaris group and 3 (14%) in the control group. Protruding malapposed struts were observed in one control patient but no Magmaris patients. Table 4 lists the quantitative OCT parameters for both groups at follow-up and for patients who underwent OCT at both baseline and follow-up (enabling comparison of the paired measurements, as with the QCA data above). Neoatherosclerosis was apparent in 7 of 15 (47%) patients in the Magmaris group and 10 of 21 (48%) patients in the control group.

**Table 3:** OCT data

OCT data at the 12-month follow-up in both groups			
	Magmaris group (*n* = 14)	Control group (*n* = 21)	*P*-value
Proximal ref. area (mm^2^)	7.72 ± 1.96	8.08 ± 2.83	0.34
Proximal ref. diameter (mm)	3.10 ± 0.39	3.17 ± 0.55	0.35
Distal ref. area (mm^2^)	6.27 ± 1.69	7.26 ± 2.14	0.12
Distal ref. diameter (mm)	2.80 ± 0.39	3.0 ± 0.44	0.13
Mean lumen area (mm^2^)	6.62 ± 2.47	6.95 ± 1.70	0.45
Mean lumen diameter (mm)	2.82 ± 0.51	2.95 ± 0.35	0.32
Minimal lumen area (mm^2^)	4.09 ± 1.55	5.19 ± 1.16	*0.03*
Minimal lumen diameter (mm)	2.24 ± 0.39	2.59 ± 0.38	*0.009*
Magmaris group (paired comparisons, *n* = 14)			
	Baseline OCT	Follow-up OCT	*P*-value
Proximal ref. area (mm^2^)	7.94 ± 2.24	7.72 ± 1.96	0.29
Proximal ref. diameter (mm)	3.14 ± 0.44	3.10 ± 0.39	0.30
Distal ref. area (mm^2^)	6.45 ± 1.69	6.27 ± 1.69	0.75
Distal ref. diameter (mm)	2.83 ± 0.41	2.80 ± 0.39	0.73
Mean lumen area (mm^2^)	7.63 ± 1.44	6.62 ± 2.47	0.14
Mean lumen diameter (mm)	3.10 ± 0.29	2.82 ± 0.51	0.07
Minimal lumen area (mm^2^)	6.36 ± 1.30	4.09 ± 1.55	< *0.001*
Minimal lumen diameter (mm)	2.83 ± 0.29	2.24 ± 0.39	< *0.001*
Xience group (paired comparisons, *n* = 9)			
	Baseline OCT	Follow-up OCT	*P*-value
Proximal ref. area (mm^2^)	8.61 ± 3.09	7.81 ± 3.06	0.24
Proximal ref. diameter (mm)	3.25 ± 0.59	3.12 ± 0.56	0.38
Distal ref. area (mm^2^)	6.76 ± 2.16	7.19 ± 2.80	0.39
Distal ref. diameter (mm)	2.79 ± 0.38	2.97 ± 0.57	0.48
Mean lumen area (mm^2^)	7.73 ± 1.43	6.73 ± 1.65	*0.006*
Mean lumen diameter (mm)	3.11 ± 0.28	2.91 ± 0.35	*0.010*
Minimal lumen area (mm^2^)	6.3 ± 1.64	5.1 ± 1.31	*0.014*
Minimal lumen diameter (mm)	280 ± 0.38	2.58 ± 0.32	*0.012*

*ref* reference

**Table 4:** Causes, timings, and types of clinical events in both groups

Group	Type of event	Time of event	Clinical condition and comments
Control	TLF, edge-restenosis	5 months	Unstable angina
Magmaris	TLF, restenosis (neoproliferation)	10 months	Unstable angina
Magmaris	TLF, restenosis (strut collapse)	7 weeks	Unstable angina
Magmaris	Stent thrombosis	6 weeks	MI, DAPT discontinuation by patient
Magmaris	TLF, restenosis (strut collapse)	3 months	Unstable angina

*TLF* target lesion failure, *MI* myocardial infarction, *DAPT* dual antiplatelet therapy

### Imaging Data from Patients Experiencing Clinical Events

**Fig. 2:**
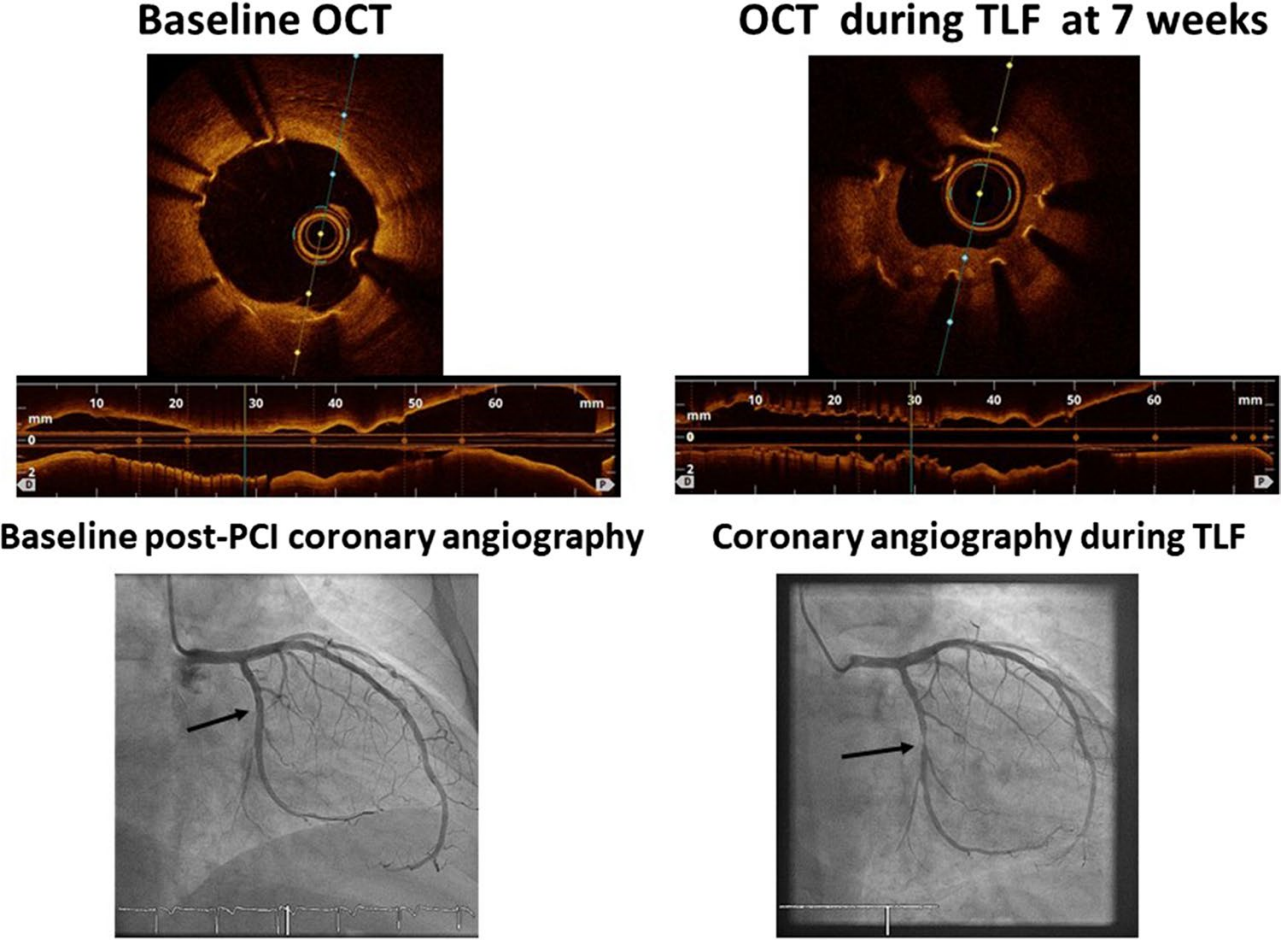
Angiographic and OCT images obtained after percutaneous cardiac intervention in a patient with a Magmaris stent (left) and early target lesion failure caused by strut collapse (right)

**Fig. 3:**
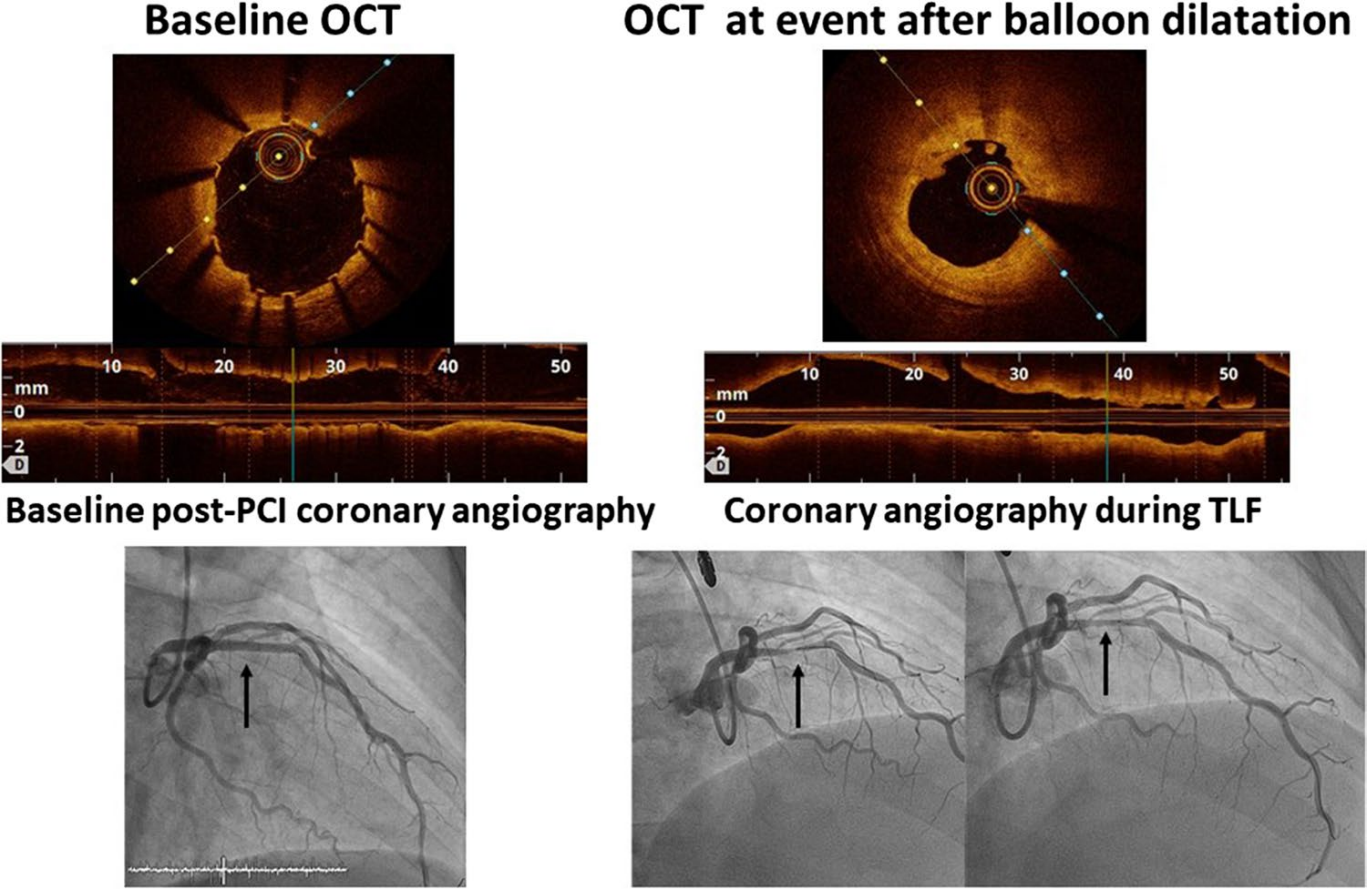
Angiographic and OCT images obtained after percutaneous cardiac intervention in a patient with a Magmaris stent (left) and target lesion failure at 10 months after the procedure (right). Tight angiographic restenosis is evident in the images on the right. OCT performed after balloon predilation of the stenosis revealed neoproliferation and stent collapse without malpositioning

During follow-up, three clinical events (all unstable angina) that required target lesion re-intervention and one stent thrombosis (associated with dual antiplatelet therapy discontinuation) occurred in the Magmaris group. One clinical event (unstable angina) that required target lesion re-intervention occurred in the control group. Table 4 shows the details. Patients with clinical events were treated with either ticagrelor or prasugrel, except for the patient with a stent thrombosis who had stopped taking clopidogrel 14 days before the event. The thrombosis was treated via thrombus aspiration and drug-eluting stent implantation. OCT was not performed during this procedure. Two patients in the Magmaris group exhibited early recurrence of angina symptoms and underwent coronary angiography; in-stent re-stenoses were apparent. Stent collapse with strut malpositioning and discontinuities were found on OCT of one patient in the Magmaris group (Fig. 2), who was then treated with a drug-eluting metallic stent implantation. Neoproliferation and stent collapse was seen in one patient in the Magmaris group who developed unstable angina symptoms at 10 months (Fig. 3). High-level stent-edge neoproliferation without stent collapse was observed in one control patient with target lesion failure (TLF).

## Discussion

We evaluated the utility of a magnesium-based bioresorbable stent in the setting of acute coronary syndrome. Our principal findings were that the extent of late lumen loss at the 12-month follow-up was significantly greater in the Magmaris group, complete resorption at 12 months postimplantation was evident in 60% of the Magmaris patients, and clinical events seemed to be associated with strut collapse in the Magmaris group.

Greater late lumen loss was experienced by the Magmaris group despite optimal implantation featuring high rates of both pre- and postdilation. Acute Magmaris stent recoil was not in play. The baseline angiographic data revealed similar acute residual stenosis rates of 7% in the Magmaris group and 10% in the control group. Furthermore, optimal final results after Magmaris implantation were confirmed via OCT in 23 of 25 patients. Similar findings in terms of late lumen loss were noted after Magmaris-stent use in the MAGSTEMI trial [8].

We noted advanced bioresorption at 12 months in approximately two-thirds of the patients with Magmaris stents, as did the OCT MAGSTEMI substudy [9]. Advanced bioresorption was unfortunately associated with vessel recoil in some segments and vessel remodelling of other segments. Neoatherosclerosis (fibrocalcified plaques near vessel lumina) may reflect preexisting atherosclerotic processes that became more visible after stent bioresorption. In both groups, the neoatherosclerotic changes mostly involved mildly fibrocalcified plaques.

In terms of clinical events, one case of stent thrombosis was probably attributable to antiplatelet medication nonadherence. Also, although data on the Magmaris stent are fewer than those for the Absorb stent, it appears that thrombosis is not a major issue with the Magmaris stent. This was confirmed in the Biosolve-IV trial; definitive/probable Magmaris stent-associated thromboses occurred in only 5 of 1075 (0.5%) patients [6]. Based on our imaging data and in vitro studies of two bioresorbable stents, we hypothesize that the high, acute radial force of the Magmaris stent better embeds the struts in vessel walls [11, 12]. However, the fast resorption and short length of scaffolding trigger more late lumen loss. These mechanisms also probably explain the stent collapses in patients with early clinical events. Our TLF rate after Magmaris-stent implantation was very similar to that of the MAGSTEMI trial (16.2% of the patients required target lesion revascularization). However, our TLF rate was almost threefold that of the BIOSOLVE-IV trial. Such discrepancies support the current European Society of Cardiology recommendation that bioresorbable stents should not be used other than in well-controlled clinical studies [13].

### Limitations

The major limitations of our study are the small number of patients, the lack of calculation of a required sample size, and the incomplete imaging follow-up at 12 months (only baseline OCT data were available for some patients). However, most studies on Magmaris stenting in acute coronary settings also included a limited number of patients (the largest study, the MAGSTEMI trial, enrolled 150), and invasive follow-up data are seldom reported [14, 15]. Furthermore, at the time of study commencement, no data on imaging assessment of Magmaris stents in the acute coronary setting were available; thus, we could not calculate the required sample size. Our work should be viewed as a pilot randomized study that precedes future adequately powered studies.

## Conclusion

Both QCA and OCT revealed a greater extent of late lumen loss in the Magmaris group than in the Xience group. Advanced bioresorption and the short length of scaffolding explain the strut collapses observed in patients with clinical events. Our data confirm the results of the MAGSTEMI trial that angiographic efficacy was lower after Magmaris stent than drug-eluting metallic stent placement in patients with acute coronary syndrome.

### Impact on Daily Clinical Practice

Magnesium-based bioresorbable stents are not appropriate for routine clinical use in acute coronary settings. The extent of late lumen loss is considerable despite optimal implantation. Further improvements are needed.

## Data Availability

The data that support the findings of this study are not openly available because they are human data but are available from the corresponding author upon reasonable request (author location: University Hospital Kralovske Vinohrady, Prague, Czech Republic).
